# Determination of ED50 and time to effectiveness for intrathecal hydromorphone in laboring patients using Dixon’s up-and-down sequential allocation method

**DOI:** 10.1186/s12871-018-0603-8

**Published:** 2018-10-05

**Authors:** Vikas O’Reilly-Shah, Grant C Lynde

**Affiliations:** Department of Anesthesiology, Emory University School of Medicine, 1364 Clifton Road NE, Atlanta, GA 30322 USA

**Keywords:** Hydromorphone, Analgesics, Opioid, Labor, Obstetric, Intrathecal

## Abstract

**Background:**

With the increasing occurrence of drug shortages, understanding the pharmacokinetics of alternative intrathecal opioid administration has gained importance. In particular, additional data are needed to comprehensively evaluate the analgesic properties of intrathecal hydromorphone in the laboring patient. In a phase 2 clinical trial, we set out to determine the median effective dose (ED_50_) and time to effectiveness for this drug in this population.

**Methods:**

Using Dixon’s up-and-down sequential allocation method, twenty women presenting for labor analgesia were prospectively enrolled. A combined spinal-epidural technique was used to deliver the determined dose of intrathecal hydromorphone. Visual analog pain scores were obtained assessing peak pain scores during serial uterine contractions. Effective pain relief was defined as achieving a pain score of less than or equal to 3 out of 10. The dose was deemed to be ineffective if the patient failed to achieve this level of relief after 30 min.

**Results:**

The ED_50_ of hydromorphone in our population was 10.9 μg (95% confidence interval 5.6–16.2 μg). Amongst patients for whom the dose was effective, the median time to pain relief was 24 min. One patient experienced both nausea and pruritus. No other complications were noted.

**Conclusion:**

Due to the prolonged time to onset, hydromorphone cannot be recommended in favor of substantively better alternatives such as sufentanil and fentanyl.

**Trial registration:**

Clinicaltrials.gov registration number: NCT01598506.

## Background

Spinal delivery of opioid analgesics is an important tool in the armamentarium of the anesthesiologist to provide analgesia while reducing side-effects from local anesthetics. The limited number of preservative-free options suitable for intrathecal injection, combined with increasingly frequent drug shortages and changes in pricing, has increased the importance of understanding the suitability of various agents [1–3]. Labor analgesia presents unique challenges due to the frequency and amplitude of changes in pain level that accompany uterine contractions, and techniques continually evolve to promote successful management of pain and reduction in breakthrough pain [4].

Fentanyl and sufentanil are lipophilic and have been established as rapidly efficacious for labor analgesia [5]. Morphine has been found to be efficacious for postoperative analgesia following Caesarean delivery, but it has been found to have a limited role in labor analgesia because of its slow onset due to its relative lipid insolubility [6, 7]. One recently published study has questioned the value of hydromorphone as an effective adjunct to intrathecal bupivacaine for labor analgesia [8]. The study had limitations, however, because the authors chose 20 min as their endpoint when maximal analgesia may not be achieved until later.

In the present work, we used Dixon’s up-and-down sequential allocation method to investigate the ED_50_ of hydromorphone in a phase 2 clinical trial [9–11]. We have previously reported the ED_50_ of hydromorphone for postoperative analgesia in Caesarean delivery and identified a value significantly lower than what was commonly assumed [12]. Our primary goal was to reject the null hypothesis that hydromorphone is ineffective for labor analgesia as measured by dose and time to onset. Additionally, we wanted to identify the median onset time in those patients who experienced relief.

## Methods

Institutional IRB approval (IRB#54701) and an FDA IND (115523) were obtained. The study was registered in clinicaltrials.gov (NCT01598506). Per protocol, written informed consent was obtained from all subjects enrolled in the study. From January 2013 to June 2014, twenty women presenting for labor analgesia were enrolled. All women in labor with cervical dilation ≥3 cm and experiencing pain greater than 5/10 during contractions who did not meet exclusion criteria were considered for enrollment. Exclusion criteria included difficulty understanding English, ASA PS status of 3 or greater, cervical dilation > 7 cm, category 2 or 3 FHT, known fetal anomaly, prior laparotomy, greater than two prior Cesarean deliveries, contraindication to neuraxial analgesia, allergy or hypersensitivity to hydromorphone, severe liver or kidney impairment, administration of opioids or sedating agents while in labor, or severe respiratory disease.

A combined spinal-epidural was placed at the L1/2 or L2/3 interspace using a 17 g Touhy needle using loss of resistance (LOR) to normal saline. Subsequent to LOR, a 25 g Whitacre needle was inserted into the dura and confirmed with free flow of cerebrospinal fluid (CSF). The CSF was aspirated and 2 mL solution containing normal saline and the study medication was injected. The initial dose of hydromorphone injected was 12 μg. If a patient reported less than or equal to 3/10 pain during contractions at 30 min, the subsequent dose was reduced by 2 μg. If a patient reported greater than 3/10 pain during contractions at 30 min, the subsequent dose was increased by 2 μg.

The CSF was aspirated again at the end of injection. An Arrow FlexTip Plus catheter (Teleflex Inc., Morrisville, NC, USA) was then inserted into the epidural space. The catheter was not tested or bolused until after the 30-min pain score was assessed. All patients had continuous FHR monitoring throughout the study period. Patients were asked about potential side effects by the anesthesiology team, including pruritus, nausea, and sedation at one hour and one day following delivery. Additionally, patients were monitored for side effects including hypotension, respiratory depression, and fetal bradycardia for twelve hours post-injection.

### Statistical methods

Using Dixon and Massey’s methodology and equation, the ED_50_ was calculated using the series of up and down sequentially allocated doses. Amongst patient for whom the drug was effective, the median time to effectiveness was calculated.

## Results

**Fig. 1:**
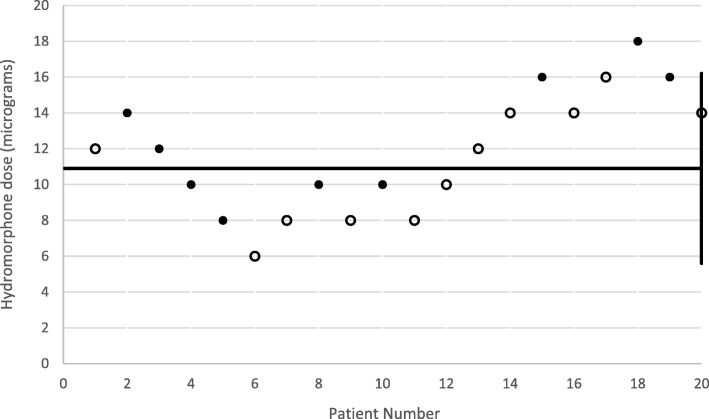
Scatter plot demonstrating dosages reported as effective (solid) and ineffective (hollow) for all 20 participants. The horizontal line represents the ED50 and was determined to be 10.9 mcg (95% CI +/−1.2 mcg)

Table 1 demonstrates baseline characteristics of the twenty women enrolled in this study. Nine reported pain scores ≤3/10 during contractions at 30 min. These women reported experiencing pain relief between range 14 and 29 min post-injection. Using the Dixey and Massey’s methodology, the ED_50_ was determined to be 10.9 μg (95% confidence interval 5.6–16.2 μg) (Fig. 1). Only one patient experienced nausea and pruritus within one hour. No other patients experienced side effects. One baby had a one-minute Apgar score less than 7 and all babies had five-minute Apgar scores of 8 or greater. None required intubation or ventilation greater than five minutes.

**Table 1:** Characteristics of Study Participants. Data are mean (standard deviation) or median [interquartile range]

Characteristic	Success	Not-success
Age	26.1 (3.5)	24.9 (3.8)
Race		
White	0	0
Black	8 (88.9%)	10 (90.9%)
Hispanic	1 (11.1%)	1 (9.1%)
Body mass index (kg*m2)	30.6 (7.7)	29.9 (6.4)
Parity		
Nulliparous	0	0
Primiparous	2 (22.2%)	5 (45.5%)
Multiparous	7 (87.8%)	6 (54.5%)
Gestational age (weeks)	38.1 (2.5)	38.8 (2.4)
Cervical dilation (cm)	4.1 (25–75% Interquartile range 3.5–5)	4.4 (25–75% Interquartile range 3–5)
Baseline visual analogue pain score	7.9 (1.0)	9.2 (1.0)
Any baseline nausea, sedation, or pruritis	0	0

## Discussion

The dose range for the ED_50_ of hydromorphone for labor analgesia is consistent with what was previously reported for Cesarean delivery [12]. The median time to pain relief in successful administrations was 24 min (range 14–29 min). These findings are consistent with, and expand on, previously reported data with respect to the role of intrathecal hydromorphone in the management of labor pain. The decision to use 3/10 reported pain was made because it is considered to be the top of “mild” and a level above which patients may not feel that their analgesia is adequate.

The time to onset is a significant limitation to using hydromorphone for controlling labor pain. Most patients demand rapid relief, which intrathecal injection of local anesthetic and/or rapid-onset opioids such as fentanyl and sufentanil which are able to provide. For this reason, routine use of hydromorphone without the addition of local anesthetic cannot be recommended by the authors.

Mhyre and colleagues were unable to conclude that 100 μg intrathecal hydromorphone significantly reduced intrathecal bupivacaine requirement for labor analgesia [8]. That group measured effective reduction in pain scores within 20 min but conceded that the 20 min window may have been too short. Concordantly, in our population, only two of the nine subjects that experienced relief achieved that relief in under 20 min.

We have previously reported that, for post-Cesarean pain, the median effective hydromorphone dosage was 5.6 μg (± 1.8 μg) [12]. In contrast, the ED_50_ for labor pain is nearly twice as high. This is likely due to a combination of factors. First, the post-Cesarean study included adjuncts (acetaminophen and ketorolac) as part of the pain management regimen. Secondly, labor pain is periodic and highly acute which is likely to require higher receptor occupancy for effectiveness [13].

In this cohort of 20 patients, only one participant experienced nausea and pruritus, side effects commonly associated with intrathecal opioid administration [14]. Others have previously reported that reductions in the dose of intrathecal hydromorphone can reduce the incidence of these side effects [15]. Although our study was not designed or powered to statistically assess these side effects, the ability to rationally approach the dosing of intrathecal hydromorphone using the ED_50_ of the drug should help to mitigate side effects by exposing patients to only the minimal necessary dose.

In the United States, intrathecal use of hydromorphone is off-label per licensing by the United States Food and Drug Administration. We and others have discussed concerns about lack of demonstrated safety of intrathecal hydromorphone, specifically as it relates to neurotoxicity [8, 12, 16–18]. However, given the long track record of safety of other opioids (sufentanil, fentanyl, morphine, and meperidine), we are reasonably reassured with respect to hydromorphone [19].

A few limitations to the present work should be noted. First, we did not control for cephalopelvic disproportion, shoulder dystocia, or other factors that might lead to greater difficulty in the management of labor pain. Second, our patient population were exclusively of black or Hispanic descent. However, we do not expect there to be racial differences in sensitivity to hydromorphone as it is not a prodrug. Additionally, while understanding the ED_50_ does provide a data point for understanding the therapeutic dosing range of a medication, the ED_50_ will be lower than the clinically useful dose of a medication that would be administered for labor analgesia. We also did not design the study to allow for the assessment of the prolonged effects of hydromorphone on the subsequent effectiveness of labor analgesia, an effect seen when using morphine and described by Vasudevan et al. Finally, it should be noted that the ED_99_/ED_95_ cannot be reliably determined based on the ED_50_, and further work is needed to define to optimal dose [20].

## Conclusions

Due to the prolonged time to onset, hydromorphone cannot be recommended in favor of substantively better alternatives such as sufentanil and fentanyl. In situations where no alternative agents are available, small quantities of local anesthetic may be added to the intrathecal injection, providing a more rapid onset of action as well as better sacral coverage than epidural local anesthetic injection [21, 22].

## Abbreviations

CSF: Cerebral spinal fluid
ED_50_: Median effective dose for 50% of the population
LOR: Loss of resistance
SE: Standard error
